# Mandatory Physical Education Classes of Two Hours per Week Can Be Comparable to Losing More than Five Kilograms for Chinese College Students

**DOI:** 10.3390/ijerph17249182

**Published:** 2020-12-08

**Authors:** Dawei Bao, Zixiang Xiao, Yuanyuan Zhang, Gang Chen, Xinyu Miao, Bo Wang, Jing Li, Chi Xu, Shuqing N. Teng

**Affiliations:** 1 Department of Physical Education, Nanjing University, Nanjing 210023, China; baodawei851219@126.com (D.B.); chengang121@nju.edu.cn (G.C.); 2 School of Life Sciences, Nanjing University, Nanjing 210023, China; 161140062@smail.nju.edu.cn (Z.X.); zhang_yyyuan@163.com (Y.Z.); miaoxy@smail.nju.edu.cn (X.M.); bowang@smail.nju.edu.cn (B.W.); jing.li.mail99@gmail.com (J.L.)

**Keywords:** physical inactivity, physical fitness tests, within-subject effects, muscular endurance, institutional interventions, sex differences

## Abstract

Despite releases of governmental guidelines for promoting physical fitness among the youth in China, the performance of college students in fitness tests has been declining over the past three decades. Obesity and physical inactivity have been proposed as two main causes. However, their relative importance for improving physical fitness remains unclear. To address this knowledge gap, we collected longitudinal data spanning four consecutive years on the physical fitness test for students from Nanjing University, China. Physical education classes of two hours per week were mandatory for the first two years. Using mixed effects models, we quantify the within-subject effects of weight, muscular endurance, sex, and mandatory physical education courses, among other variables, on physical fitness total score. We found that, in spite of the dominance of normal weight among the students, losing weight was positively associated with the total score, with significant sex differences in the associations. Compulsory exercise provided by physical education classes per week had strong positive impacts on the total score, comparable to losing weight of roughly 15–17 kg for males and 5–10 kg for females. Half sex difference in the total score was explained by male students’ poor performance in the muscular endurance represented by pull-ups. Our results suggest that college students in China should engage in physical activity of higher levels to improve their physical fitness, with a heightened awareness of extra fat under normal weight and insufficient muscular endurance.

## 1. Introduction

Rapid economic growth and urbanization since China’s reform and opening-up in the late 1970s have induced great changes in many aspects of the society. Despite improved nutrient supply and quality, the health-related physical fitness, usually assessed in combination of body composition, cardiorespiratory endurance, flexibility, and muscular strength and endurance, of contemporary Chinese students at the age of 7 to 22 has considerably declined compared to that in the 1980s, imposing worrying challenges to national public health [1,2]. To improve the fitness of young generations, the Chinese government proposed in 2007 that children in primary and adolescents in secondary school, commonly 7 to 18 years old, should participate in physical exercise for at least one hour per day [3]. Since 2010, likely due to this nationwide call, the physical fitness of school-aged children and adolescents has leveled off [1] and started to rebound [2]. In the recent “Healthy China 2030” blueprint [4], children and adolescents are further expected to master at least one sport and engage in physical activity of moderate to vigorous intensity for at least three times per week, with 25% of the population meeting the excellent level of physical fitness. On the other hand, the physical fitness of Chinese college students commonly at the age between 19 and 22 did not seemingly benefit from this intervention targeted at them prior to college enrollment but continued to decline without signs of leveling off over the past three decades [2,5].

The contrasting responses of physical fitness between the two age groups of Chinese students beg the question of whether top-down interventions that aim to promote physical activity and thus improve physical fitness would be less effective for students in the transition from adolescence to adulthood. Indeed, the majority of adolescents older than 15 years have been reported to fall short of the recommended one hour per day of moderate to vigorous physical activity [6], with further declines in physical activity levels for young adults in their twenties [7]. The effectiveness of such interventions seems to diminish with the age of students. As behavior patterns established during the adolescence-to-adulthood transition can have long-term effects on health and career [8,9], it is important for government agencies and educational institutions to develop effective guidelines and regulations for fitness improvement among college students, conditional on a good understanding of their fitness deterioration.

Largely because college students have greater autonomy in their daily lives than during secondary education, they are more subject to weight gain and developing unhealthy behaviors such as physical inactivity and poor diet [10,11]. Importantly, weight gain and unhealthy behaviors are usually intertwined with each other, making it difficult to assess which risk factor is dominant in the declining of physical fitness. For example, overweight and obese students are closely associated with sedentary behavior, unbalanced dietary structure, and excessive caloric intake [12]. Among self-regulated behaviors, weight management has been suggested as an important approach to weight problems among college students [13]. As both individual motivation and environmental cooperation are emphasized for successful weight management [13], the effectiveness of behavior change remains unclear due to the complexity of confounding factors [14]. Currently, there is no consensus on the optimal effective approach to arresting the deterioration of physical fitness among college students.

Physical education has long been considered as a feasible top-down channel to deliver recommended physical activity and improve fitness among the youth [15]. Although the positive relationship between physical education and physical activity promotion has been well demonstrated [16,17], the degree to which physical education can improve fitness and health is still open to debate [16,18]. It is important to note that, due to differences in the quality of physical education, many physical education programs have been reported to miss the recommended minimum level of moderate to vigorous physical activity [19], compromising the potential for fitness improvement among young people. In addition, academic load for college undergraduates can marginalize their effort devoted to physical education courses that are often offered as optional, with the expected fitness benefits unrealized [20].

Given that the relative importance of weight management and physical education remains unclear, evidence-based interventions and guidelines are urgently needed for both short-term fitness improvement and long-term fitness maintenance among the population of college students. Here, using longitudinal data on college students’ physical fitness covering the entire study span from 2015 to 2019 in Nanjing University, China, we aim to examine the dynamics of their performance in multiple physical fitness tests and the weighted total fitness score, investigate the associations among these tests and the total score, and further quantify the effect sizes of weight and mandatory physical education courses on the total score. Our findings can quantitatively inform future institutional and national guidelines for improving the physical fitness of young adults. 

## 2. Materials and Methods

### 2.1. Subjects and Their Physical Fitness

The subjects in this study were the undergraduate students from Nanjing University enrolled in the year of 2015. The Department of Physical Education, Nanjing University, annually conducted a series of health-related tests to assess the physical fitness of each subject, with written informed consent obtained from the mentors on behalf of the subjects prior to data collection. The subjects took the tests every year from 2015 to 2018. Following the terminology [21] for health-related research, these tests involved body composition (here represented by height and weight), cardiorespiratory endurance (here represented by vital capacity of lung and middle-distance running), flexibility (here represented by sit-and-reach), muscular strength (here represented by standing long jump and 50 m dash), and muscular endurance (represented by male-specific pull-ups, female-specific sit-ups, male-specific 1000 m, and female-specific 800 m middle-distance running). 

The study was conducted in accordance with the Declaration of Helsinki, and the protocol was approved by the Institutional Review Board of Nanjing University (DPHAE (2014) No. 4).

According to the criteria published by the Ministry of Education of the People’s Republic of China for assessing the physical fitness of Chinese students [22], the original value from each test, with weight and height combined as Body Mass Index (BMI; weight in kilograms divided by the square of height in meters), was converted to a grade out of 100 points. The total score on the same grading scale for each subject was then recorded using the formula: total score = 0.15 × BMI + 0.15 × vital capacity + 0.2 × 50 m dash + 0.1 × sit-and-reach + 0.1 × standing long jump + 0.1 × pull-ups (or sit-ups) + 0.2 × middle-distance running. Considering that the records for the middle-distance running test were subject to data quality issues due to inconsistent grating decisions among examiners in the case of many test retakes, we computed another total score on a scale of 1 to 80 points excluding the middle-distance running test from the formula above.

In addition to data derived from these physical fitness tests, other information related to the subjects were provided. There were 3202 subjects in total involved in the tests four years in a row, who were from 31 provincial-level administrative divisions across China and affiliated to 28 departments and schools: 1039 of them were from the Jiangsu province, with 2006 from the other 30 divisions and 157 not recorded for their origin. Although a few subjects transferred once from their original department on enrollment to another department in the first or second year, no extra combinations of subject and department other than the original combinations on enrollment were recorded in the dataset. Throughout the four years, 11,346 entries of data were generated, with 5989 from males and 5357 from females. 

Physical education courses mandatory for completing a bachelor’s degree were offered by the university in the first two years of the four-year education program. The courses, scheduled for two hours per week in each semester, were on general physical education for the first semester in the first year and one specific sport for each of the following three semesters. Students may take specific sports among basketball, volleyball, soccer, badminton, table tennis, tennis, shuttlecock kicking, Chinese martial arts, tae kwon do, gymnastics, yoga, orienteering, swimming, Ultimate Frisbee, and track and field. Optional physical education courses were offered in the third and fourth years. 

### 2.2. Variable Selection

As the total score we computed in this study is a linear combination of each score on BMI, vital capacity, sit-and-reach, standing long jump, 50 m dash, and male-specific pull-ups or female-specific sit-ups, accounting for all of them in regression modeling will result in perfect collinearity. Instead, we first used a scatterplot matrix to explore the correlations among the total score and the single tests. The single tests that were both correlated with the total score and other single tests were used as explanatory variables for modeling, without suffering from perfect collinearity and missing variable bias [23]. As the significance test for small coefficients that are of no practical significance tends to be statistically significant in a large sample [24], we chose the absolute value of correlation coefficient > 0.3 [25] as the threshold of being practically significant in this study. In particular, we focused on identifying physical fitness tests that are correlated with weight with practical significance.

To account for the potential effects of local socioeconomic status before university enrollment on the fitness test scores, the gross domestic product (GDP) per capita in 2015, of the hometown of each subject, was retrieved at the prefectural city level from the 2016 statistical yearbooks for the 31 provinces [26]. Admission into Nanjing University is highly selective on a provincial basis, with candidates from prefectural cities under each provincial division competing for performance in sciences and liberal arts. Without the pressure of physical fitness competition in the National College Entrance Examination (NCEE), many families and high schools in China choose to compromise on the amount of time for physical exercises and outdoor sports so as to allocate more resources to compulsory NCEE examinations. Therefore, we expected that prefectural expenditures for promoting residents’ physical activity should be irrelevant to the physical fitness of subjects who had top NCEE performance in this study.

### 2.3. Model Selection

Before enrollment into the university, the students were nurtured in localities where they took NCEE. Apart from individual genetic factors, their physical health and fitness prior to enrollment were to varying degrees related to family and societal factors, such as family socioeconomic status, after-school program participation, local neighborhood, physical education in school, and recreational and sports facilities [27]. However, detailed information on each subject’s background and experiences were inaccessible within the scope of the physical fitness test. Given heterogeneity bias caused by between-subject differences [28], such above-mentioned individual, family, and societal factors cannot be simply treated as random effects in mixed-effects models when assessing the *ceteris paribus* effects (i.e., the causal or partial effects when other variables are controlled for) is the purpose [29]. To avoid heterogeneity bias and at same time estimate the coefficients of nesting binary variables (i.e., the sex in this study), we adopted the approach of mixed effects regression modeling that accounts for between-subject effects, instead of fixed effects regression modeling, to assess the within-subject effects of explanatory variables [30].

Our unbalanced longitudinal data spanning four years was structured in three levels. Each test record per year at level one was nested within the 3202 subjects at level two, which were nested within the 28 departments and the 31 provinces at level three, with the departments and provinces as partially crossed factors [31]. Due to the immaturity of modeling partially crossed random effects where one cross-classified factor is missing for some observations [32], 157 subjects whose origin province was absent in 441 data entries were excluded from regression modeling. The resulting 10,905 data entries were analyzed with mixed-effects models, where the specification of random effects followed the suggestion for the alternative scenario of partially crossed random effects [32]. 

To quantify the effects of weight and mandatory physical education courses on physical fitness, the fixed effects of the mixed-effects models included the physical test variables as selected above, a dummy variable denoting the presence or absence of physical education courses in the first or latter two years, a dummy variable representing the male or female sex, as well as interaction terms between the sex variable and the other fixed-effects variables. The random effects of the fixed-effects models consisted of student IDs as the lowest level nested within the two cross-classified province and school levels, as well as errors for each observation. The structure of the mixed-effects models was as follows:

Total score (of 80 points) ~ Weight + Other correlated physical fitness tests + Exposure to Physical education classes + Sex + Interaction terms with sex + Nested subject-level and crossed department- and provincial level random effects + Independent and identically distributed errors.

As we are interested in the explanatory role of weight and physical education classes in physical fitness scores rather than predicting the score from these variables, the Bayesian Information Criterion (BIC) was used for model comparison [33].

All statistical analyses were in R (version 3.4.4) [34] with the *lme4* package [35].

## 3. Results

**Figure 1:**
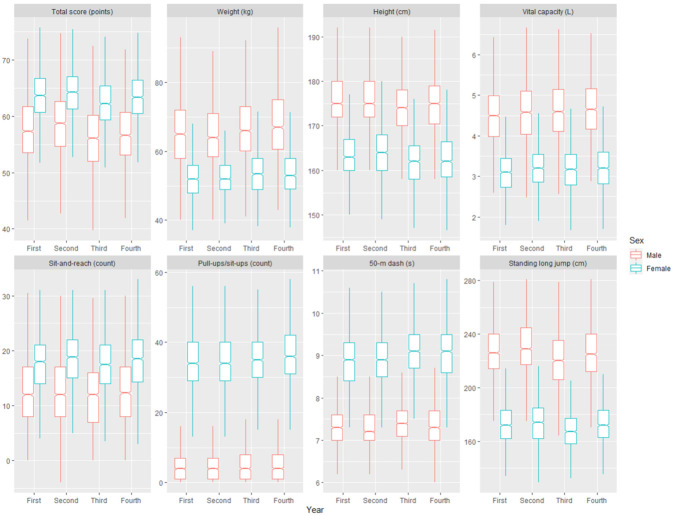
Boxplots of the total score and numerical measurements of seven single tests for four years in a row from 11,346 records for the 3202 subjects (red for male and blue for female). Middle line in box denotes the median. Notches in box represents a roughly 95% confidence interval for the median. Non-overlapping notches indicate significantly different at the 0.05 level. Outliers are not shown. Physical education was scheduled in the first and second years but absent in the third and fourth years.

For both male and female subjects, the median total scores in the second year were significantly (*α* = 0.05) higher than those in the other three years, sharing the same pattern with the single test of standing long jump (Figure 1). The median male weight was the lowest in the same year, with their speed for the 50 m dash being the fastest. In contrast, the median female height was the greatest in this year, with lighter weight and faster 50 m dash but fewer sit-ups for the first two years (Figure 1). Male subjects’ performance in sit-and-reach and pull-ups remained stable throughout the four years, while their vital capacity increased monotonously (Figure 1).

**Figure 2:**
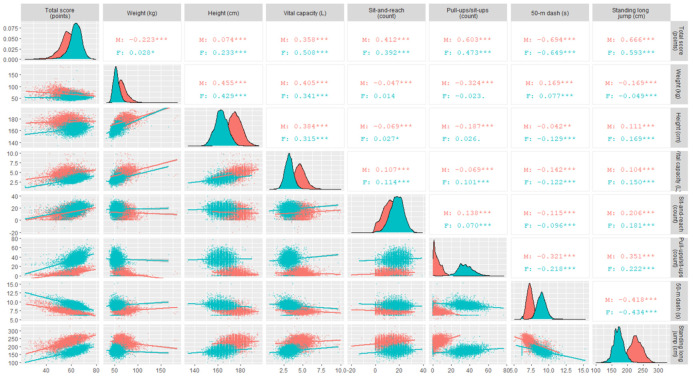
Scatterplot matrix of the total score and numerical measurements of seven single tests from 11,346 records for the 3202 subjects (red for males (M) and blue for females (F)), with their units in parenthesis and their density curves on the diagonal. The lower triangle shows the scatter plots of one variable on the y axis against another variable on the x axis, with linear regression line for each sex. The upper triangle shows the Pearson correlation coefficients between these variables, with three, two, and one stars, and one dot indicating statistical significance at the *α* = 0.001, 0.01, 0.05, and 0.1 level, respectively. Correlation coefficients with an absolute value >0.3 meet the criterion for variable selection in this study (see Materials and Methods Section).

In terms of the linear correlation with the total score, all single tests were significant (*α* = 0.001 except for the female weight at *α* = 0.05) for each sex, with 50 m dash and standing long jump presenting large (absolute value >0.5) [25] coefficients for both sexes (Figure 2). Among the seven single tests, the three correlation coefficients for each pair between weight, height, and vital capacity tests and the correlation coefficient between the two muscular strength tests (i.e., 50 m dash and standing long jump tests) were practically significant (coefficient absolute value >0.3) for both male and female subjects (Figure 2). The count of male-specific pull-ups had practically significant correlations with weight, 50 m dash, and standing long jump, while the count of female-specific sit-ups had no practical importance for other tests (Figure 2). Therefore, we included weight, height, vital capacity, and male-specific pull-ups as four explanatory variables in regression modeling.

As shown in Table 1, the between-subject and within-subject effects of weight and pull-ups differed under all scenarios, with height and vital capacity being less so for males, confirming the presence of heterogeneity bias and thus supporting the estimates of within-subject effects. For an individual subject of either sex, with all other things being equal, a one-kilogram gain in weight would result in a roughly 0.1 decrease in the total score of physical fitness, and a 100-milliliter increase in vital capacity would generally mean a 0.37 increase in the total score. Increasing one centimeter in height corresponded to an increase of 0.11–0.12 in total score points for males and 0.05–0.06 for females. Performing one more pull-up would add 0.7 points to the total score of male subjects. For subjects from all provinces, the physical fitness of females scored 12 points higher than that of males, with this difference being five points larger for subjects from the Jiangsu province and 2–4 points smaller for those from other regions. Physical education courses had a positive impact of 1.9–2.0 and 1.1–1.2 points on the total score for males and females respectively, with 0.4 points less for subjects from the Jiangsu province.

## 4. Discussion

Our four-year longitudinal data showed that female students enrolled at Nanjing University in the year of 2015 performed consistently better than males in terms of the total score of annual physical fitness tests, in line with our quantitative analysis where the female sex represented more than 10 score points in comparison to the male sex. Several studies on the physical fitness of Chinese students have reported similar findings. For example, female freshmen from another top university in China have outperformed male freshmen in almost every single score of physical fitness tests for five years in a row [36]. Chinese male students prior to higher education have also presented a lower pass rate of physical fitness tests than that of the female population [37]. The underlying reasons for the distinction of physical fitness scores between the two sexes remain to be explored (see also the discussion below).

Between-subject heterogeneity of physical fitness resulted mainly from individual background and behavior. Both parental and personal factors have been reported to be significantly associated with the motivation of Chinese students for physical activity and exercise [5,38], thus important for physical health and fitness among the population. Variances in physical fitness at the provincial and department levels were not negligible in this study, as model performance would decline without accounting for them in random effects. At a broader regional scale, the differences in single physical fitness tests among students across China have been noted with no clear explanations [36].

Within-subject changes in the weight, height, and vital capacity played different roles in affecting the total score of physical fitness. Specifically, weight had an overall consistent negative within-subject effect, with strong province-dependency (−0.1542 vs. −0.0951) for females. BMI values greater than 23.9 and 27.9 were defined as overweight and obesity respectively, for both sexes [22], resulting in a negative relationship between weight and physical fitness for this BMI interval. However, the majority, i.e., 72.76% (= 4175/5738) for males and 87.88% (= 4541/5167) for females, of the records in this study were within the interval of normal weight (Table 2). Furthermore, only 8.84% (= 507/5738) male and 4.20% (= 217/5167) female records were in the fringe BMI interval between 22.9 and 23.9 (Table 2), where degeneration from normal weight into overweight would be more likely, suggesting that statistical signals of decreasing scores in response to increasing weight would be difficult to be detected. It is therefore reasonable to expect that the negative within-subject coefficients of weight should not be closely related to BMI-based overweight and obesity. It has been reported that students under normal weight obesity (BMI between 18.5 and 23.9 kg/m^2^ and fat percentage greater than 20%) were associated with poorer physical fitness, partially due to less skeletal muscle mass [39], suggesting that increases in body fat of normal weight students would compromise physical fitness. Potential functional links between Chinese college students’ weight and physical fitness remain to be proposed and tested. On the other hand, as there were no obvious distinctions of BMI frequency distribution between female subjects from and not from the Jiangsu province, the province-dependency of within-subject effects of female weight remains unexplained.

Both height and vital capacity had positive within-subject effects on physical fitness scores. Given the magnitude of differences in median weight, height, and vital capacity between the first and latter two years (Figure 1), their net within-subject effects on physical fitness scores would be more or less canceled out. Importantly, male subjects had extremely poor performance in pull-ups with a consistent median of four rated less than 10 score points, while the pass count rated 60 score points is 10 and 11 for the first and latter two years, respectively [22]. In comparison, the median of female-specific sit-ups was 34, 35, and 36 for the first two, the third, and the last years respectively, rated between 68 and 70 score points [22]. To catch up the performance gap in muscular endurance between the two sexes, male subjects would have to complete 8–9 more pull-ups to meet the rating of 68 score points [22]. This improvement would result in an increase of roughly six (averaged from 0.7 × 8 and 0.7 × 9) points for the total score of males, clearing half the score lag behind females (Table 1). As another muscular endurance test, the middle-distance running, was excluded from our analysis due to concerns about data quality, the role of muscular endurance in explaining the distinction of physical fitness scores between the two sexes cannot be further explored. In fact, the data quality concern for the middle-distance running test is a strong reflection of the worrying situation in which the muscular endurance of Chinese college students of both sexes has experienced the most noticeable decline over the past three decades [5], indicating the immediate necessity of promoting aerobic exercise.

Compared with weight, height, and vital capacity, mandatory physical education classes had a much stronger positive impact on physical fitness total scores, especially for the male sex (Table 1). Specifically, mandatory physical education of two hours per week was comparable to weight loss of more than 15 (2.006/0.1229 = 16.32; 1.874/0.1146 = 16.35; 1.595/0.0944 = 16.90) kilograms for males and more than 5 (0.858/0.1542 = 5.56; 1.093/0.1078 = 10.14; 1.218/0.0951 = 12.81) kilograms for females. This quantitative finding is consistent with the marked distinction of total score between the first and latter two years in Figure 1, with significant associations between physical education classes and several single physical fitness tests. A recent survey has found that 80% of male and 89% of female college students in China spent less than one hour per day in physical activity [38], with self-reported decreasing energy expenditure in daily life during the four-year higher education program [40]. As academic credits for physical education courses are compulsory for graduation from Nanjing University, it is reasonable to expect that the subjects must have allocated more time to physical activity in the first two years than in the latter. The strong positive relationship between mandatory physical education courses and physical fitness score may be closely linked to body composition and muscular strength, given that there were obvious differences between the second and third years in terms of weight, height, 50 m dash, and standing long jump (Figure 1). Stronger enthusiasm among Chinese male students for physical activity [38,41] may indicate that males could better benefit from mandatory physical activity, probably explaining the sex difference in the effect size of physical education classes (Table 1).

Our rigorous regression analysis of rich longitudinal data on the fitness of students from Nanjing University provides quantitative evidence that mandatory physical education classes are more effective than simple weight control in terms of physical fitness improvement. To arrest the lasting fitness decline among Chinese college students over the past three decades, placing physical education into a much less marginalized position in comparison to conventional core academic subjects should be under serious consideration of government agencies and higher education institutions in China. In fact, the Ministry of Education of China announced in 2019 that college students who fail physical fitness tests shall not meet the standard for graduation [42]. In 2020, the Central Office and the State Council General Office of China jointly issued a guideline to deepen educational reform in which physical education escalates as an equally important subject assessed in NCEE, and within- and outside-curriculum physical activity for two hours per day is warranted with an aim to help young generations develop lifelong active behavioral patterns [43].

One limitation of our study is that, due to data quality issues in the middle-distance running test, we were unable to comprehensively assess the role of muscular endurance in physical fitness of college students. The contribution of aerobic exercise within- and outside-curriculum remains to be further explored. Besides, the small within-subject effects of weight in our study can only be interpreted as low effectiveness of simple weight control. As demonstrated by other studies, scientific and effective weight management involving complex strategies for college students is a cost-effective opportunity to form healthy lifestyle patterns among adults [13,14].

## 5. Conclusions

Our study found that the physical fitness of male students from Nanjing University was lagging behind that of females, with sex-specific negative effects of weight and positive effects of height and vital capacity. This lag can be partially explained by the poor performance of male students in the muscular endurance represented by pull-ups. Dynamics of weight, height, and vital capacity during the span of four years canceled out on average their respective impacts on physical fitness scores. Physical education classes of two hours per week can significantly improve the physical fitness of both sexes, comparable to losing more than 15 kg for males or more than 5 kg for females. Our study supports that interventions consisting of mandatory physical activity and effective aerobic exercise should be considered as the priority for keeping Chinese college students in fitness.

## Figures and Tables

**Table 1:** Output from the mixed-effects models selected by Bayesian Information Criterion. Estimates of intercept (Intcpt; in points), weight (W; in kilograms), height (H; in centimeters), vital capacity (VC; in liters), pull-ups (P; a count for males (M) only), exposure to mandatory physical education classes (C) of two hours per week, state of being female (F), and their interaction terms in the best mixed-effects models under nine scenarios. 3045 subjects in the regression modeling were comprised of 1039 from the Jiangsu province (J) and 2006 from 30 other provincial regions (Non-J). Gross domestic product (GDP) per capita of home prefectural cities was not in the pool of selected fixed terms under all scenarios. Between-subject effects represent the group-level score point responses to a unit change in the tests, while within-subject effects represent the individual-level score point responses to a unit change in the tests.

Origin	Sex	Intcpt.	Between-Subject Effects							Within-Subject Effects							Dummy Variables		
			W	H	VC	P	W × F	H × F	VC × F	W	H	VC	P	W × F	H × F	VC × F	C	C × F	F
All	Both	21.45	−0.1694	0.1269	4.002	1.015	-	-	1.607	−0.1120	0.0911	3.730	0.7092	-	-	-	1.921	−0.8909	11.39
	M	20.83	−0.1633	0.1311	3.910	1.027	-	-	-	−0.1146	0.1210	3.682	0.7093	-	-	-	1.874	-	-
	F	32.50	−0.1832	0.1304	5.743	-	-	-	-	−0.1078	0.0536	3.832	-	-	-	-	1.093	-	-
J	Both	20.68	−0.1671	0.1190	4.499	1.032	-	-	-	−0.1080	0.1017	3.846	0.6736	-	-	-	1.593	−0.7345	17.29
	M	21.12	−0.1625	0.1278	4.009	1.028	-	-	-	−0.0944	0.1364	3.783	0.6770	-	-	-	1.595	-	-
	F	38.68	−0.1693	0.0967	5.450	-	-	-	-	−0.1542	0.0590	3.991	-	-	-	-	0.858	-	-
Non-J	Both	20.34	−0.1715	0.1348	3.969	1.014	-	-	1.785	−0.1133	0.0828	3.690	0.7228	-	-	-	2.071	−0.9307	10.75
	M	20.97	−0.1645	0.1317	3.864	1.025	-	-	-	−0.1229	0.1104	3.653	0.7215	-	-	-	2.006	-	-
	F	29.00	−0.1893	0.1493	5.940	-	-	-	-	−0.0951	0.0476	3.768	-	-	-	-	1.218	-	-

**Table 2:** Data records by Body Mass Index (BMI) categories and sex. Underweight corresponds to BMI less than 17.8 for males and less than 17.1 for females. Normal weight corresponds to BMI between 17.9 and 23.9 for males and between 17.2 and 23.0 for females. For both sexes, overweight corresponds to BMI between 24.0 and 27.9, with BMI greater than 28.0 for obesity. The critical interval is BMI between 22.9 and 23.9, in which transition from normal weight into overweight is more likely.

Sex	Underweight	Normal Weight	Critical Interval	Overweight	Obesity	Total
Male	402	4175	507	900	261	5738
Female	296	4541	217	277	53	5167
